# Exploring Patients’ Perceptions About Chronic Kidney Disease and Their Treatment: A Qualitative Study

**DOI:** 10.1007/s12529-023-10178-x

**Published:** 2023-05-24

**Authors:** Yvette Meuleman, Yvonne van der Bent, Leandra Gentenaar, Fergus J. Caskey, Hans AJ. Bart, Wanda S. Konijn, Willem Jan W. Bos, Marc H. Hemmelder, Friedo W. Dekker

**Affiliations:** 1 Department of Clinical Epidemiology, Leiden University Medical Centre, Leiden, the Netherlands; 2 Population Health Sciences, University of Bristol, Bristol, UK; 3 Dutch Kidney Patients Association, Bussum, the Netherlands; 4 Department of Nephrology, Leiden University Medical Centre, Leiden, the Netherlands; 5 Department of Internal Medicine, St Antonius Hospital, Nieuwegein, the Netherlands; 6 Department of Internal Medicine, Maastricht University Medical Centre, Maastricht, the Netherlands; 7 CARIM School for Cardiovascular Research, University Maastricht, Maastricht, the Netherlands

**Keywords:** Chronic kidney disease (CKD), Illness perceptions, Patient-reported outcome measures (PROMs), Person-centered healthcare, Qualitative research, Self-regulation theory

## Abstract

**Background:**

Unhelpful illness perceptions can be changed by means of interventions and can lead to improved outcomes. However, little is known about illness perceptions in patients with chronic kidney disease (CKD) prior to kidney failure, and no tools exist in nephrology care to identify and support patients with unhelpful illness perceptions. Therefore, this study aims to: (1) identify meaningful and modifiable illness perceptions in patients with CKD prior to kidney failure; and (2) explore needs and requirements for identifying and supporting patients with unhelpful illness perceptions in nephrology care from patients’ and healthcare professionals’ perspectives.

**Methods:**

Individual semi-structured interviews were conducted with purposive heterogeneous samples of Dutch patients with CKD (*n* = 17) and professionals (*n* = 10). Transcripts were analysed using a hybrid inductive and deductive approach: identified themes from the thematic analysis were hereafter organized according to Common-Sense Model of Self-Regulation principles.

**Results:**

Illness perceptions considered most meaningful are related to the seriousness (illness identity, consequences, emotional response and illness concern) and manageability (illness coherence, personal control and treatment control) of CKD. Over time, patients developed more unhelpful seriousness-related illness perceptions and more helpful manageability-related illness perceptions, caused by: CKD diagnosis, disease progression, healthcare support and approaching kidney replacement therapy. Implementing tools to identify and discuss patients’ illness perceptions was considered important, after which support for patients with unhelpful illness perceptions should be offered. Special attention should be paid towards structurally embedding psychosocial educational support for patients and caregivers to deal with CKD-related symptoms, consequences, emotions and concerns about the future.

**Conclusions:**

Several meaningful and modifiable illness perceptions do not change for the better by means of nephrology care. This underlines the need to identify and openly discuss illness perceptions and to support patients with unhelpful illness perceptions. Future studies should investigate whether implementing illness perception-based tools will indeed improve outcomes in CKD.

**Supplementary Information:**

The online version contains supplementary material available at 10.1007/s12529-023-10178-x.

## Introduction

With the ongoing shift towards person-centred healthcare, increased attention is paid towards the perceptions that patients hold [1]. A growing body of literature suggests that especially patients’ illness perceptions are key to understanding why many patients with chronic conditions (e.g. diabetes mellitus and cardiovascular disease) have poor health outcomes that cannot be explained by the clinical severity of the condition alone [2–4]. Illness perceptions are part of Leventhal’s Common-Sense Model (CSM) of Self-Regulation: this model proposes that when people are faced with a health threat (e.g. diagnosis or symptoms), this evokes cognitive and emotional perceptions, and these perceptions help people to make sense of the situation they are confronted with (i.e. how serious and controllable is the disease). These illness perceptions also affect how patients respond to and cope with the disease (e.g. treatment adherence [e.g. adopt a healthy lifestyle or take medication as prescribed], seek support, etc.) and subsequently contribute to health outcomes [2, 3, 5, 6].

A large body of literature has shown that illness perceptions of patients with kidney failure are associated with various outcomes, including depression, health-related quality of life (HRQOL) and mortality [e.g. 7, 8]. Until now, few studies have focused on earlier stages of chronic kidney disease (CKD), but the longitudinal studies that have been conducted suggest that strong negative illness perceptions are common and a marker for poor outcomes [9–11]. For example, an accelerated disease progression (i.e. a faster kidney function decline and an earlier start of dialysis) and increased odds for an unfavourable HRQOL-trajectory were detected in patients who attributed more symptoms to their CKD; believed to a lesser extent that they fully understand their CKD and can personally control their CKD; and believed to a higher extent that their CKD has negative consequences upon their lives, has an unpredictable cyclical nature and causes emotional distress [9, 10].

Furthermore, studies have shown that unhelpful illness perceptions can be changed by means of psychoeducational support strategies and can lead to improved coping behaviours and health outcomes [12–14]. Hence, identifying unhelpful illness perceptions may create unique opportunities to improve patient-reported and clinical outcomes in patients prior to kidney failure. Currently, no tools exist in routine nephrology care to identify and support patients with unhelpful illness perceptions. To build adequate, timely and personalized assessment and support tools, in-depth knowledge is needed about (the story behind) illness perceptions (e.g. which illness perceptions underlie patients’ personal experiences and ability to cope with CKD and how these illness perceptions evolve over time) and about stakeholders’ needs regarding illness perception-based tools. Therefore, this study aims to identify from the patients’ and healthcare professionals’ perspectives: (1) meaningful and modifiable illness perceptions and (2) needs and requirements regarding identifying and supporting patients with unhelpful illness perceptions prior to kidney failure. By not only including patients’ perspectives but also that of professionals, insight into the topics is enriched by their years of experiences caring for patients with CKD and will facilitate the development of tools that fit routine nephrology care.

## Method

### Design and Participants

Individual face-to-face semi-structured interviews were conducted between January and October 2019 in the Netherlands. Purposive sampling ensured a heterogeneous patient-sample representing the CKD population (e.g. regarding gender, age, educational level and comorbidities) and a heterogeneous professional-sample representing diverse occupations (e.g. nephrologist, nurse practitioner, social worker and dietician). Eligibility criteria for patients were: ≥ 18 years old, sufficient understanding of the Dutch language, estimated glomerular filtration rate of ≤ 30 ml/min/1.73 m^2^ (CKD stage 4–5: the stages in which the kidneys are severely damaged and getting close to failure) and currently not receiving dialysis treatment. Professionals were eligible when involved in the care for patients with CKD. Professionals were recruited from Leiden University Medical Centre (LUMC) via the research team, and patients via their LUMC care team and via the National and Regional Hollands-Midden Kidney Patients Associations (Nierpatiënten Vereniging Nederland and Diavaria). Recommended guidelines and checklists (e.g. COnsolidated criteria for REporting Qualitative research [COREQ]) were used to conduct and report this study [15, 16].

### Study Procedure

An interview-protocol was developed based on literature [e.g. 2, 3, 5, 6], to maintain consistency in the interviews’ format. All study documents were discussed with stakeholders (patients, professionals and representatives of Kidney Patients Associations), 5 pilot-interviews were held with members of Kidney Patients Associations, and documents were adapted based on feedback and pilot-experiences. Patient-interviews consisted of: 1) ‘think-aloud’ assignment in which patients spoke aloud while filling out two commonly-used, validated illness perception questionnaires (i.e. Brief and Revised Illness Perception Questionnaires [B-IPQ; IPQ-R]) [17–20], hereby gaining insight into patients’ personal thoughts about their illness underlying a question, reasons for specific answers, and which questions did (not) correspond with their experiences; and 2) semi-structured interview focusing on stakeholders’ perspectives regarding: illness perceptions underlying patients’ experiences, outcomes and coping abilities; most meaningful and modifiable illness perceptions; and needs and requirements regarding identifying and supporting patients with unhelpful illness perceptions. Professional-interviews consisted of the semi-structured interview including a discussion about the existing B-IPQ/IPQ-R-questionnaires [19, 20]. The interviewer followed the topic-list’s structure (Supplementary File 1), but deviated from it when appropriate. All questions were open-ended and responses were further explored using additional questions and probes. Interviews were conducted by one investigator (an experienced interviewer and psychologist, trained to conduct interviews as part of this qualitative research), recorded digitally (audio recordings using a professional Olympus voice recorder), and field notes on dynamics and nonverbal communication were taken. Participants completed a brief questionnaire to collect participant characteristics.

### Analysis

Interviews were transcribed verbatim and reviewed line-by-line by three investigators. Transcripts were not returned to participants for feedback; however, to ensure that participants’ responses were correctly understood (e.g. also when it comes to emotions and non-verbal communication), a verbal summary of topics and main issues discussed was given at the end of each interview, and participants were invited to respond to this summary and indicate whether this summary was correct (and if not, provide corrections). A hybrid inductive and deductive approach was used for analysis: (1) inductive phase: transcripts were analysed using thematic analysis (i.e. descriptive analytical method to identify, organize and provide insight into patterns in qualitative data [i.e. themes] using constant comparison, grouping and hierarchically organizing themes) [21, 22]; and (2) deductive phase: identified themes were now organized according to CSM-principles [5, 6]. Initial coding and analysis was done by one investigator in close collaboration with a second investigator (two experienced qualitative researchers, knowledgeable in the field of healthcare and psychosocial aspects of chronic [kidney] disease). To judge consistency of interpretation, both investigators coded two transcripts of patients and professionals, and codes were compared and discussed. During the analysis process, interpretations were iteratively reviewed and critically discussed until consensus was reached. Data saturation was continuously evaluated, until the agreement was reached that no new information was obtained and themes emerged [23]. To ensure triangulation [16], a multidisciplinary research team was composed to conduct this study, consisting of diverse perspectives and experiences (psychology and medical students, psychologists, patient-representatives, epidemiologists, nephrologists; 4 females and 5 males), with expertise in qualitative research, nephrology care and psychosocial aspects of CKD. Transcriptions were coded using ATLAS.ti v8 (GmbH). An audit trail was kept, and all files were saved on a secured server. Finally, illustrative quotes were selected and translated from Dutch to English using back translation.

## Results

Twenty-seven interviews were conducted (17 with patients and 10 with healthcare professionals) for a mean duration of 71 ± 20 min. Interviews predominantly took place in LUMC-meeting rooms and four interviews at patients’ homes in the regions Hollands-Midden and Friesland. Patients’ partners were present during two interviews. Table 1 shows all participant characteristics. As shown in Table 2, mean IPQ-R/B-IPQ-scores often laid around the scales’ midpoint. Exceptions include: patients believed to a high extent that their CKD is chronic in nature, that they understand their CKD and that their treatment can effectively control their CKD. Below, the main findings are shown within the context of the CSM-principles, structured following the study aims and presented with illustrative quotations.

**Table 1:** Characteristics of patients with CKD and healthcare professionals (*n* = 27)

	**Patients** ***n* = 17**	**Professionals** ***n* = 10**
**Sex**, *n* (%) male	12 (70.6)	3 (30.0)
**Age**, mean ± *SD* years	64.9 ± 13.7	53.1 ± 8.8
**Marital status**, *n* (%) married/partnered	8 (47.1)	8 (80.0)
**Ethnicity**, *n* (%) Dutch	13 (76.5)	8 (80.0)
**Highest level of education**, *n* (%)^a^		
Primary education	1 (5.9)	
Secondary education	8 (47.1)	
Vocational education	1 (5.9)	
Tertiary education (college/university)	4 (23.5)	10 (100)
**Work status**, *n* (%)^a^		
Full-time	2 (11.8)	7 (70.0)
Part-time	2 (11.8)	3 (30.0)
No — Home/retired	7 (41.2)	
No — Disabled due to other reasons	3 (17.6)	
**Kidney transplantation**, *n* (%)	1 (5.9)	
**Time since CKD diagnosis**, mean ± *SD* years	15.6 ± 12.1	
**Comorbidity**, *n* (%)	15 (88.2)	
Diabetes mellitus	4 (23.5)	
Cardiovascular disease	4 (23.5)	
Other	7 (41.2)	
**Frequency hospital visits**, mean ± *SD* times per year with nephrologist-internist and/or nurse practitioner	6.1 ± 2.2	
**Healthcare profession**, *n* (%)		
Nephrologist		5 (50.0)
Nurse practitioner		2 (20.0)
Dietician		2 (20.0)
Social worker		1 (10.0)
**Involved in CKD treatment**, median years (*IQR*)		16.6 (10.1)

Data presented as mean ± standard deviation (*SD*) for normally distributed continuous variables and as median with interquartile range (*IQR*) for skewed continuous variables. Count (percentage) was used for categorical variables

^a^Data on education level and work status available for 14 patients (82.4%)

**Table 2:** Illness perception scores of patients with CKD (*n* = 17)^a^

**Illness perception**	**IPQ-R**^b^ Mean ± *SD* on 1–5 scale	**B-IPQ**^c^ Mean ± *SD* on 0–10 scale	**A higher score indicates that patients believe to a greater extent that…**
Illness identity	4.0 ± 2.7	5.2 ± 2.7	…their CKD causes more symptoms
Timeline acute/chronic	4.2 ± 0.5	9.4 ± 1.4	…their CKD lasts for a longer time
Cyclical timeline	2.6 ± 0.9	*N.A*	…their CKD and related symptoms have an unpredictable cyclical nature
Personal control	2.6 ± 0.8	6.9 ± 2.7	…their CKD can be effectively controlled by themselves
Treatment control	2.1 ± 0.6	8.8 ± 2.6	…their CKD can be effectively controlled by their treatment
Illness coherence	3.0 ± 0.6	8.6 ± 2.7	…they understand their CKD
Consequences	3.2 ± 0.8	6.2 ± 2.2	…their CKD has more negative consequences upon their life
Emotional response	2.6 ± 0.8	4.8 ± 2.7	…their CKD causes more emotional distress
Illness concern	*N.A*	5.8 ± 3.2	…their CKD causes greater worries

^a^Like other studies assessing illness perceptions amongst patients with CKD, illness perception ‘cause’ was not included due to the heterogeneous causes of CKD. For all illness perception-scores (IPQ-R and B-IPQ), a higher score means a stronger illness perception.

^b^The following IPQ-R domains were assessed using 38 questions on a 5-point Likert scale: timeline acute/chronic, cyclical timeline, consequences, personal control, treatment control, illness coherence, and emotional response. The domain ‘illness identity’ addressed different physical symptoms attributed to CKD and was measured using 14 items in a yes or no format. Domain scores were created following the official IPQ-R instructions.^19^

^c^The B-IPQ assessed the same illness perceptions as the IPQ-R with the exception that illness perception ‘concern’ was measured instead of ‘timeline cyclical’. All eight illness perceptions were measured by means of a single item on a 11-point scale.^20^

### Meaningful Illness Perceptions in Patients with CKD Prior to Kidney Failure

**Fig. 1:**
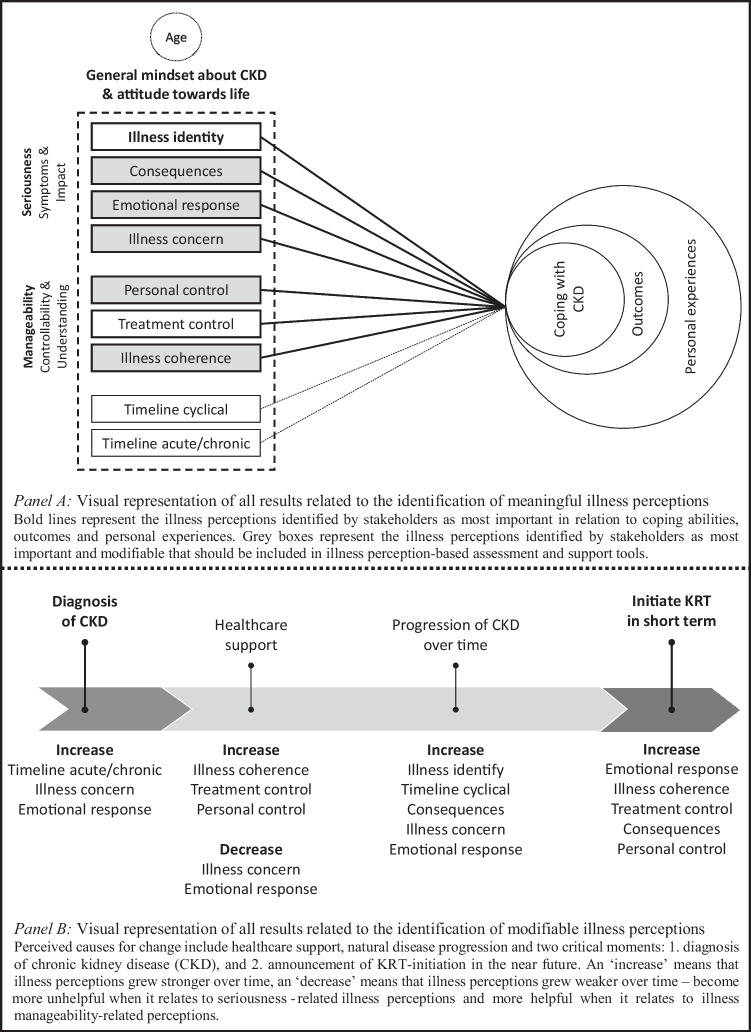
Visual representation of all results related to the identification of meaningful illness perceptions (**A**) and modifiable illness perceptions (**B**) in patient with CKD prior to the initiation of kidney replacement therapy (KRT)

Figure 1A shows a visual representation of all results related to the identification of meaningful illness perceptions.

#### Illness Perceptions Underlying Coping, Outcomes and Experiences

Patients and professionals believed that all illness perceptions underlie patients’ experiences and coping abilities. The illness perceptions considered most important were either related to the seriousness of CKD (beliefs about the condition’s symptoms and its impact: illness identity, consequences, emotional response and illness concern) or the manageability of CKD (beliefs about controllability and patients understanding of the condition: personal control, treatment control and illness coherence).

##### Seriousness-Related Illness Perceptions: Symptoms & Impact of CKD

Patients and professionals considered ‘illness identity’ to be crucial. They explained that some patients do not experience symptoms of CKD and, hence, do not consider themselves a patient — they only see their kidneys as ‘diseased organs’ and experience CKD through consultations:

I only experience my illness through test results on the computer screen. Besides that, I have no idea that my kidneys are failing. (Patient/male/78years[y])

Not experiencing symptoms greatly influences patients’ experiences and coping behaviours (e.g. not adhering to treatment guidelines). Both patients and professionals shared that patients who *do* experience CKD-related and/or treatment-related symptoms also experience the many negative consequences of CKD upon their lives (‘consequences’), strongly influencing patients’ experiences, coping abilities and other illness perceptions. For example, fatigue can cause patients to experience limitations in their ability to participate in social activities (‘consequences’), which can evoke strong negative feelings such as sadness (‘emotional response’). Patients considered one consequence to be particularly important, namely ‘uncertainty about the future’: not knowing how rapid kidney function will decline, when and which kidney replacement therapy (KRT; transplantation, dialysis, or conservative management) needs to be initiated etc., which prevents patients from making plans for the future and fuels their concerns (‘illness concern’):

I think that's the worst: that you know it [KRT] is coming but not when. (Patient/female/69y)

There's a silent killer in this [CKD]. You don't know exactly what will happen. (Patient/male/67y)

##### Manageability-Related Illness Perceptions: Controllability & Understanding of CKD

Patients and professionals stressed that ‘personal control’ was essential for patients’ experiences, outcomes and coping abilities:

When you have more control, you feel empowered. Then people generally feel better – they feel they have control over the situation. (Nephrologist)

Patients stated that they (and many other patients) want to contribute as much as possible to slow down CKD progression: adopt a healthy lifestyle, monitor disease progression, read up on CKD pathophysiology and treatment-developments etc. Both patients and professionals believed that patients can contribute to temporarily stagnate disease progression (e.g. by adequate self-management). However, both acknowledged that treatment non-adherence is common and that kidney function can still deteriorate, even when patients perfectly adhere to treatment guidelines, which caused patients to experience a lack of control:

You can drink a lot and you can limit salt and protein intake, but I think that's just a drop in the ocean. (Patient/woman/24y)

Given this difficulty to control actual disease progression, patients considered it crucial that professionals focused to a greater extent on improvement of patients’ experiences:

You cannot really control disease improvements, except for the usual medical treatment. Improvement is mainly improvement in the experience. [….] you can do a lot about that. (Patient/male/85y)

Professionals considered ‘treatment control’ especially important for patients’ experiences and coping abilities: patients need to trust their doctor, treatment and healthcare; and this could be achieved by having a good doctor-patient relationship and practicing evidence-based medicine — hereby ensuring optimal treatment and realistic expectations. Patients and professionals believed ‘illness coherence’ was important as well, especially for patients’ coping abilities and to strengthen patients’ control perceptions (‘treatment and personal control’):

I think it’s really connected: if you don’t understand the disease, you can’t control it. (Nurse practitioner).

Personal control provides an incentive to delve more deeply into your illness. And better insight means better behavior. (Social worker)

#### General Mindset About CKD and Attitude Towards Life

Patients and professionals felt that all illness perceptions are intertwined, together forming patients’ general mindset about CKD (‘how serious and manageable is this condition’) that influences patients’ experiences, coping and outcomes (e.g. disease progression and HRQOL). They stated that an overall positive perception of CKD and being hopeful and optimistic in life is most beneficial:

How you deal with it [CKD], has mainly to do with your mindset. With a little bit more positivity […] you see that the glass is half full. If not, you should take a smaller glass. (Patient/male/51y)

It depends on your attitude towards life. I truly believe that, if you have a positive attitude towards life, you get much further than you would expect based on physical condition. (Nephrologist)

For some patients, this meant they needed to learn how to mentally disconnect from CKD sometimes, while others shared they remain hopeful by focusing on the possibility of kidney transplantation. Professionals stated that an overall positive attitude was most often seen in older patients, with possible explanations being: (1) younger patients experience greater consequences because they still work, take care of their children and have a future ahead of them; and (2) older patients have more life experiences, which positively influences their coping abilities:

If you’re 70 or 75, it’s just as impactful and just as bad. But older people can put things into perspective and say: ‘Yes it’s terrible, but I sat in the waiting room next to someone aged 32’. (Nurse practitioner)

### Modifiable Illness Perceptions in Patients with CKD Prior to Kidney Failure

Patients and professionals believed that all illness perceptions can evolve over time. Figure 1B shows a visual representation of all results related to the identification of modifiable illness perceptions. They identified two critical moments influencing trajectory-development, with the first being ‘receiving the diagnosis CKD’. Professionals stated that patients are often in shock after receiving the diagnosis. Patients shared that, initially, they were very concerned (‘illness concern’) when receiving the news they had (to live with) this chronic, progressive disease (‘timeline acute/chronic’). The latter caused patients to experience various negative emotions (‘emotional response’):

That changes over time, thankfully. I very consciously tell patients: ‘It’s a rollercoaster, the first few months’. (Nurse practitioner)

Over time and by the received healthcare, concerns and emotional responses diminished as patients learned more about the disease (‘illness coherence’) and how they themselves and the treatment can manage disease progression (‘personal control’ and ‘treatment control’):

Now that I know that I can get a transplant, concerns are less. I have been very concerned. (Patient/ male/51y)

As disease progresses, patients experienced a steady increase in CKD-related symptoms (‘illness identity’ and ‘timeline cyclical’) and, consequently, an increase in ‘illness concern’, ‘consequences’ and ‘emotional response’. Fatigue was perceived as the most impactful symptom:

The most prominent and limiting symptom is ‘fatigue’. As a result, patients will notice limitations in their daily life or reject activities to be able to keep doing at least some things. (Nephrologist)

The second moment was the announcement that ‘KRT needs to be initiated in the near future’. Professionals and patients shared that providing information and discussing KRT-options (‘illness coherence’ and ‘treatment control’) is experienced as confrontational and evokes negative emotions (‘emotional response’). Professionals added that after dialysis but also after kidney transplantation, patients still experience great distress (‘emotional response’) due to the high symptom- and treatment burden (‘illness identity’) and its negative impact (‘consequences):

The most crucial moment is starting dialysis. The impact that dialysis has on your life….. (Nephrologist)

Patients added that it also worked as incentive to pursue a healthier lifestyle (‘personal control’):

I already had that sense of personal control, but I only started acting on it when I received ‘the call’ and realized this was not going well…. (Patient/male/62y)

Finally, patients and professionals believed that support from the environment and from professionals can positively impact the illness perceptions’ development (see ‘Needs and requirements for supporting patients with unhelpful illness perceptions’).

### Needs and Requirements for Identifying Illness Perceptions in Routine Nephrology Care

#### Needs for Identifying Illness Perceptions

Patients and professionals believed that patients would benefit from tools to identify illness perceptions and that it would facilitate a stronger focus on patients’ perspectives in nephrology care. Most patients believed that all illness perceptions should be measured because the importance of illness perceptions will vary from one person to the next:

I think they are all important. It differs per person what is important to that person. (Patient/woman/24y)

Others ranked the importance: ‘timeline acute/chronic’ was considered least important because patients know CKD is a chronic condition; and ‘consequences’, ‘personal control’, ‘illness coherence’, ‘illness concern’ and ‘emotional response’ were identified as essential due to its impact on experiences and coping (see also Fig. 1A). Patients and professionals shared that prioritizing was difficult due to illness perceptions’ interconnectedness, for example, understanding CKD (‘illness coherence’) will increase personal control and will reduce anxiety and concerns (‘personal control’, ‘emotional response’ and ‘illness concern’):

Some people like it when they know everything. It gives them feelings of control. […] Ignorance causes unrest. (Nephrologist)

If people know more about their disease, they will know better how to deal with it. Consequently, their concerns will diminish. (Dietitian)

#### Requirements for Identifying Illness Perceptions

Patients and professionals believed that in order to successfully develop and implement an assessment-tool, several aspects should be taken into account. First, the tool should be brief: patients believed the IPQ-R is too long and too much overlap exists between questions. Professionals added that we must avoid patients feeling overloaded. Second, questions should be clear in the CKD-context: patients stated that some IPQ-R/B-IPQ questions were unclear, for example, ‘My treatment can control my illness’ – what is ‘my treatment’ when you receive such complex multicomponent CKD-treatment? Third, it should contain the simplest language to ensure usage in as many patients as possible. Patients believed most questionnaires (including IPQ-R/B-IPQ) require relatively high levels of health literacy, language proficiency and reading abilities:

I am fairly good at the Dutch language. People who aren’t will have problems with those [IPQ-R/B-IPQ]. (Patient/male/72y)

Four, opinions differed about how and where to complete the tool. Some patients and professionals stated that digital and at home have most advantages (e.g. offers flexibility), while others felt it would be completed more seriously on paper and in the hospital:

Putting a few crosses on questionnaires at home, everyone does this within 2 minutes while you are watching soccer or something else. In the hospital, you fill it in more seriously. (Patient/male/73y)

Most patients and professions believed the tool should be completed in absence of professionals to prevent social desirability bias, while some patients applauded completion under supervision (e.g. facilitates possibilities to ask questions). Six, all participants agreed that results need to be discussed with professionals and that follow-up is essential but challenging:

I think having such a questionnaire is fantastic, but follow-up has to be completely clear. (Nephrologist)

You shouldn’t leave it at this questionnaire, it should be a reason to adequately refer someone. (Social worker)

Finally, professionals believed strenuous efforts should be made to reach patients that would benefit most, namely those with a negative attitude towards their illness, healthcare and life:

People who are doing a great job, immediately grab questionnaires and start filling it in. […] but the group that matters most, withdraws from healthcare and avoids it. (Nephrologist)

### Needs and Requirements for Supporting Patients with Unhelpful Illness Perceptions in Routine Nephrology Care

#### Needs for Supporting Patients with Unhelpful Illness Perceptions

Patients and professionals believed that support strategies for patients with unhelpful illness perceptions are needed in nephrology care. Illness perceptions identified as most important to address are: ‘illness concern’, ‘emotional response’, ‘illness coherence’, ‘personal control’ and ‘consequences’, as they are susceptible to change *and* most closely related to patients’ experiences, outcomes and coping abilities (see also Fig. 1A and B). Patients considered addressing ‘illness concern’, and ‘emotional response’ particularly important: ‘illness concern’ by providing education and reassurance and ‘emotional response’ by creating opportunities to share and receive advice on emotions and thoughts. They stated that social *and* professional support are crucial to achieve this, because they do not always want to burden their loved ones:

Sometimes you do not want to bother your family. Outsiders, yes, they listen to you. Sometimes I tell them more than I tell my own husband or children. (Patient/female/68y)

Patients and professionals regarded addressing ‘illness coherence’ as important, with the potential to also positively impact patients’ acceptance and control-perceptions. A straightforward strategy is to provide information that fits abilities and needs of individual patients:

You have to tell the message clearly: ‘It’s so and so, and I am always there for you’. (Nurse practitioner)

Finally, patients shared they are in need of support in dealing with CKD’s impact on their lives. Discussing (coping strategies to deal with) ‘consequences’ could greatly support them:

You cannot always change the consequences, but you can change the way you deal with them. It is important to talk about it. (Patient/male/51y)

#### Requirements for Supporting Patients with Unhelpful Illness Perceptions

Patients and professionals believed several aspects should be considered to ensure successful development and implementation of strategies. First, support should consist of various (already existing) modules. Ideally, there is one module for each illness perception; patients can then choose and/or professionals can refer patients to one or multiple modules focusing on, amongst others, increasing knowledge, coping skills and psychosocial support. Patients encouraged consultations with social workers and/or psychologists to receive adequate support in coping with (consequences of) CKD, concerns and emotions:

We all have moments when you think ‘It bothers me!’. Then it is really nice if someone helps and comforts you, and afterwards you feel ‘It is all very annoying, but I can still do a lot things!’ (Patient/male/64y)

Professionals agreed that support should focus on coping strategies and emphasized professionals should always respect and strengthen patients’ autonomy:

If you push for something patients don’t want, they might do it for the doctor. That’s undesirable. Leave the choice with patients and let them know they can always reconsider their decision. (Nephrologist)

Second, opinions differed on whether support should be digital and/or physical, as preferences will most likely differ from one person to the next. Professionals suggested that physical meetings are best combined with existing hospital visits or otherwise organized outside the hospital. Third, opinions differed on whether strategies should comprise individual and/or group support. Patients believed that a disadvantage of group support is that not everyone gets the same amount of opportunities to share because some group-members always dominate the groups. An advantage of peer support is that patients share experiences and give advice, and this was considered important because patients are more likely to take on advice from and ask questions to fellow patients:

This so-called ‘peer contact’, I have noticed, it is often very useful, because people are more likely to take on advice from someone who has it [CKD] themselves. (Patient/male/64y)

Professionals stated that patients are initially often hesitant to join peer groups but often find these sessions very useful:

I don’t think anyone actually thinks ‘Oh nice, I'm going to be in such a group’. But I do think it is more useful than patients expect in advance. (Dietician)

Four, professionals stressed that strategies should not be standalone but should be structurally embedded into nephrology care. For patients, what’s most important is that they know support is out there when they need it:

People need to know it’s available. Ultimately, it’s up to them whether they want to use it. […] If you don’t use it now, that’s okay. Just know it’s there, that you can find support and information there. (Nurse practitioner)

Finally, involvement of and caring for patients’ significant others were considered crucial because CKD has a great impact on their lives and wellbeing:

Family members deal with all kinds of consequences from the disease. It is less costumery they receive support […] Caring for caregivers is something that could be improved. (Patient/male/72y)

## Discussion

This study showed that patients with CKD and healthcare professionals considered illness perceptions related to the seriousness (illness identity, consequences, emotional response and illness concern) and manageability (illness coherence, personal control and treatment control) of CKD most meaningful. They also believed that patients developed more helpful manageability-related illness perceptions and more unhelpful seriousness-related illness perceptions over time. Implementing tools to identify and discuss patients’ illness perceptions was considered important, after which support for patients with unhelpful illness perceptions should be offered.

First, the CSM of Self-Regulation appears to be a useful theoretical model to explore perceptions about CKD. We found a good fit between our results and CSM-principles, for example, patients and professionals strongly believed that illness perceptions are multidimensional, interrelated and underlie experiences and outcomes of patients with various chronic conditions [2–14]. However, contrary to the CSM that makes no explicit assumption on the relative importance of individual illness perceptions in different contexts, our participants considered some illness perceptions more important than others in the CKD-context. For example, all illness perceptions were identified as important for patients’ experiences, outcomes and coping abilities except for timeline perceptions: ‘timeline cyclical’ was almost non-existent during interviews and ‘timeline acute/chronic’ was identified as least important. These results are in line with research showing that some illness perceptions are more strongly associated with certain outcomes [7–11], for example, stronger negative perceptions of illness identity, emotional response, consequences and personal control are often associated with more distress and impaired HRQOL, while weaker perceptions of treatment control are often associated with increased mortality in CKD-populations. However, these studies also suggest that timeline perceptions play an important role in CKD outcomes (e.g. stronger negative cyclical timeline perceptions are associated with a faster kidney function decline and an earlier start of dialysis) [8–10]. A possible explanation is that our participants took it as a given that all patients believed CKD to be a chronic, progressive condition with steadily increasing symptoms over time. Such beliefs (e.g. strong chronicity and weak-to-moderate cyclical timeline perceptions) could be considered relatively accurate medical illness perceptions and problems will most likely occur when patients hold *inaccurate* illness perceptions (e.g. believe CKD to be a temporary, non-progressive condition with highly unpredictable symptoms) [24, 25]. Moreover, our participants also emphasized that dismissal of illness perceptions as ‘irrelevant’ is complex due to illness perceptions’ interrelatedness, for example, timeline perceptions are correlated with emotional response, consequences, illness identity and concern [19, 20].

Illness perceptions perceived as most meaningful are either related to the seriousness of CKD (i.e. symptoms and its impact: illness identity, consequences, emotional response and illness concern) or manageability of CKD (i.e. what do you need in order to manage: illness coherence, personal control and treatment control) [3, 5, 6]. Interesting to note is that responses of patients and professionals overlapped to a great extent, illustrating that these professionals have adequate insight into patients’ beliefs about CKD. The most striking difference is that patients considered seriousness *and* manageability illness perceptions most meaningful, while professionals predominantly emphasized the importance of manageability illness perceptions and in particular ‘treatment control’. These findings underline the need for professionals to adopt a more holistic approach to nephrology care: focus on managing CKD disease progression *and* supporting patients in coping with (the impact of) CKD [1, 26, 27].

Our results suggest that illness perceptions evolve over time: seriousness-related illness perceptions grew stronger (i.e. patients increasingly attribute symptoms to their CKD and believe that CKD has negative consequences, and causes worries and emotional distress) and so did manageability-related illness perceptions (i.e. patients increasingly believe they understand their CKD and that CKD can be effectively controlled by their treatment and by themselves). In other words, patients developed more *unhelpful* seriousness-related illness perceptions and more *helpful* manageability-related illness perceptions. Perceived causes for changes in illness perceptions include natural CKD progression, healthcare support (e.g. education) and two critical moments: receiving the CKD diagnosis and the message that KRT needs to be initiated soon. These results align with literature showing that illness perceptions not only change as a result of interventions targeting these illness perceptions but also change naturally and according to clinical status, medical treatment, newly obtained knowledge and experiences [3–6, 12–14, 28, 29]. Interesting to point out is that some illness perceptions seem to change more often than others, for example, timeline perceptions changed at one moment (e.g. chronicity beliefs grew instantly strong after receiving the CKD diagnosis), while illness concern and emotional response changed at multiple timepoints (e.g. grew stronger after receiving the CKD diagnosis and after experiencing an increase in symptoms and consequences) and fluctuated over time (e.g. diminished after receiving healthcare support).

### Clinical Implications and Illness Perception-Based Tools

Although longitudinal observational studies are needed to confirm illness perception-trajectories prior to kidney failure, our results suggest that several meaningful and modifiable illness perceptions do not change for the better by means of routine nephrology care. Consensus existed amongst our participants on the need for an assessment-tool to identify and openly discuss patients’ beliefs about CKD and their treatment, and for additional strategies to support patients with unhelpful illness perceptions, preferably as early as possible in the course of CKD. All nine illness perceptions were considered important for inclusion in tools as the importance differs from one person to the next. However, some illness perceptions were considered most essential, namely consequences, emotional response, personal control, illness concern and coherence, as these illness perceptions are modifiable *and* most closely related to patients’ experiences, outcomes and coping abilities. It was believed that nephrology care could greatly benefit from increased support for seriousness-related illness perceptions (i.e. dealing with consequences, emotions and concerns about the future), hereby adding to literature highlighting the need for additional psychosocial strategies to support patients in dealing with a chronic (kidney) disease [1, 4, 26, 27]. Moreover, nephrology care (e.g. education) already seems to positively influence manageability-related illness perceptions (i.e. control perceptions and illness coherence) but perhaps not in all patients yet (e.g. patients with limited health literacy skills), and these illness perceptions could potentially be strengthened to ensure beliefs are turned into actual behaviour (e.g. act on personal control beliefs by adopting a healthy lifestyle) [1, 30, 31].

Several illness perceptions were not selected, amongst others, because they are most likely already medically accurate illness perceptions that receive sufficient attention in routine nephrology care (i.e. treatment control and timeline beliefs). Illness identity was also not prioritized, which is striking because participants considered CKD-related symptoms essential to patients’ experience (especially fatigue) — literature confirms the latter, illustrating the high symptom burden in different CKD-stages [25–27, 32, 33]. A possible explanation might be peoples’ conviction that increased symptom burden is inextricably linked with the typical CKD-trajectory and that ‘there is nothing you can do about it’. Indeed, symptoms will increase with declining kidney function [24], but literature also suggests that many potentially treatable CKD-related symptoms often remain undiscussed and un(der)treated [34, 35]. Therefore, developing and implementing symptom-management strategies seems crucial — to reduce symptom burden and to positively impact illness perceptions (illness identity and interrelated perceptions such as consequences) and HRQOL in patients with CKD [27, 34–36]. Our study identified several requirements for illness perception-based tools. For assessment: the tool should be brief, include clear questions in the CKD-context, contain the simplest language, enable flexible completion (e.g. on paper and digitally), function as a conversation-started to openly discuss patients’ illness perceptions and be accompanied by action-plans which include support from professionals — requirements corresponding with known considerations when using patient-reported outcome measures (PROMs) in clinical practice [33, 37, 38]. For support: in line with and building on literature, a multicomponent psychosocial educational program is needed that addresses all modifiable and meaningful illness perceptions, comprises physical and digital components (N.B. interviews were held pre-COVID-19), combines individual sessions with group sessions, includes consultations with a social worker and psychologist, and incorporates peer-to-peer support (e.g. mentoring via phone and online communities) and support for caretakers [4, 12–14, 31]. Another essential requirement was that this program is no standalone program but fully embedded in nephrology care: patients need reassurance that support is out there when they need it and literature also suggests that factors such as timing, accessibility and readiness to engage should be taken into account when providing support [39, 40]. Furthermore, future studies are needed to investigate whether implementing such illness perception-based tools will indeed improve outcomes in patients with CKD (e.g. prior to kidney failure) and other chronic conditions.

Finally, our results suggest that intertwining illness perceptions form patients’ general mindsets about CKD and that a beneficial positive mindset is most often seen in older patients. These findings correspond with literature indicating that interrelated illness perceptions form patients’ illness schema [5, 6], that these so-called illness perception profiles reflect more stable dispositions towards a condition that greatly contribute towards health outcomes [41] and that positive psychological functioning (including optimism) plays an important role in adaption to and outcomes of patients with chronic conditions [42]. The role of age seems more complex: generally, average optimism levels indeed grow with age, but there are also indications that it peaks around a person’s 50/60s after which it begins to decline [43]. Until now, little is known about illness perception profiles, optimism and age-related differences in patients with CKD, and hence, additional research is warranted. Important to mention is that patients consider it challenging to stay optimistic when so much uncertainty exists about their future. The prospect of transplantation helped some patients to keep their hopes up, but not all patients are eligible for transplantation and the symptom- and treatment burden is still high after transplantation [38]. Therefore, psychosocial strategies to support patients in coping with uncertainties about the future are needed, and so are studies aimed at developing and implementing prognostic models for a broad range of patient-relevant outcomes [1, 44, 45].

### Strengths and Limitations

This study’s most important strength is that it provides unique in-depth insight into illness perceptions of patients with CKD and into how these illness perceptions evolve prior to kidney failure. By identifying meaningful and modifiable illness perceptions and by identifying needs and requirement, timely and personalized theory-based tools can be developed and implemented to identify and discuss illness perceptions, and to support patients with unhelpful illness perceptions in routine nephrology care. A study limitation is research reflectivity [16]: even though multiple investigators with different backgrounds have interpreted results, we cannot rule out the possibility that (theoretical) preconceptions have coloured results. For example, using a more socially oriented theory instead of the somewhat more cognitive and individual-focused CSM of self-regulation in the deductive phase of the analysis, could have resulted in increased insight into the role of social factors such as caretakers and significant others. Furthermore, although purposive sampling ensured a heterogeneous patient-sample representing the Dutch CKD population, transferability of our results could be improved by including more low-educated patients, more (inter)national centres, patients with earlier stages of CKD and patients with CKD since birth or their childhood. Additionally, although speculative, it is possible that participating patients may have a more positive attitude towards CKD, healthcare and life; inclusion of patients with a more negative attitude may have provided valuable information about how to reach patients that withdraw from healthcare and that would benefit most from additional support.

### Conclusion

Several meaningful and modifiable illness perceptions do not change for the better by means of routine nephrology care. This underlines the need to identify and openly discuss illness perceptions, and to provide support for patients with unhelpful illness perceptions about CKD. Special attention should be paid towards strengthening psychosocial support for patients and caregivers to deal with the many CKD-related symptoms, negative consequences and emotions, and concerns about the future.

## Supplementary Information

Below is the link to the electronic supplementary material.

Supplementary file1 (DOCX 86.3 KB)
