# Supplementary material for: Exploring Patients’ Perceptions About Chronic Kidney Disease and Their Treatment: A Qualitative Study
Source: Int J Behav Med. 2023 May 24;31(2):263–75. doi: 10.1007/s12529-023-10178-x (PMC10208195; doi:10.1007/s12529-023-10178-x)
Supplement: Supplementary file 1 — Supplementary file1 (DOCX 86.3 KB) [file 12529_2023_10178_MOESM1_ESM.docx]

**Supplementary File 1.** Interview topic guide

**Part A. ‘Think-aloud’ assignment**

Instructions to ‘think aloud’ while filling in the commonly-used and validated Brief- and Revised Illness Perception Questionnaires.^19,20^

Example follow-up questions:

- Can you tell me something about your experiences with completing the questionnaires?
- What are your thoughts about the questions? What struck you?
- Which questions did (not) correspond with your experiences?
- What made it difficult/easy for you to complete the questionnaires? Why?

**Part B. Semi-structured interview**

Important illness perceptions underlying patients’ coping abilities, outcomes and experiences

Example questions:

- I have no personal experiences with CKD; how would you explain to someone like me, what it’s like to live with CKD?
- Which thoughts come to mind when you think about CKD and your treatment?
- What happens when you think “[thought]”? How do you feel? What do you do?
- We have now discussed several thoughts, which thoughts are most important for your personal experiences and ability to cope with CKD? Why?

Development of illness perceptions prior to kidney failure

Example questions:

- How changeable do you think these thoughts about CKD and your treatment are? Why?
- How did you think about CKD and your treatment in the past? If different compared to current thoughts, what caused this change?
- How do you think your thoughts will develop in the future? Which factors (could) play a role in this development?

Assessing illness perceptions in routine nephrology care

Example questions:

- Which thoughts about CKD and your treatment are important to share with your care team? Why?
- How can measuring thoughts about CKD and your treatment contribute to your care and well-being? Why would it (not) contribute?
- What are requirements for (implementing) tools to measure thoughts about CKD and your treatment in routine nephrology care?

Support in routine nephrology care

Example questions:

- What treatment/support do you receive for your CKD?
- What kind of support do/did you need but was not provided?
- Which personal thoughts about CKD and your treatment could/should be positively influences by support? Why? Who should provide this support?
- What are requirements for (implementing) tools to positively influence thoughts about CKD and your treatment in routine nephrology care?

Interviews with professionals consisted of part B, the semi-structured interview, including a discussion about the Brief- and Revised Illness Perception Questionnaires,^19,20^ and “you” in the questions was replaced by “patients with CKD, prior to kidney failure”.
